# An Approach to Growth Delimitation of Straight Line Segment Classifiers Based on a Minimum Bounding Box

**DOI:** 10.3390/e23111541

**Published:** 2021-11-19

**Authors:** Rosario Medina-Rodríguez, César Beltrán-Castañón, Ronaldo Fumio Hashimoto

**Affiliations:** 1 Departamento de Ingeniería, Escuela de Posgrado, Pontificia Universidad Católica del Perú, Av. Universitaria 1801, San Miguel, Lima 15088, Peru; cbeltran@pucp.pe; 2 Departamento de Ciência da Computação, Instituto de Matemática e Estatística, Universidade de São Paulo, Rua do Matão, 1010, São Paulo 05508-900, SP, Brazil; ronaldo@ime.usp.br

**Keywords:** minimum bounding box, straight-line segment classifier, supervised learning

## Abstract

Several supervised machine learning algorithms focused on binary classification for solving daily problems can be found in the literature. The straight-line segment classifier stands out for its low complexity and competitiveness, compared to well-knownconventional classifiers. This binary classifier is based on distances between points and two labeled sets of straight-line segments. Its training phase consists of finding the placement of labeled straight-line segment extremities (and consequently, their lengths) which gives the minimum mean square error. However, during the training phase, the straight-line segment lengths can grow significantly, giving a negative impact on the classification rate. Therefore, this paper proposes an approach for adjusting the placements of labeled straight-line segment extremities to build reliable classifiers in a constrained search space (tuned by a scale factor parameter) in order to restrict their lengths. Ten artificial and eight datasets from the UCI Machine Learning Repository were used to prove that our approach shows promising results, compared to other classifiers. We conclude that this classifier can be used in industry for decision-making problems, due to the straightforward interpretation and classification rates.

## 1. Introduction

The computational power and the high demand for automatic systems for pattern recognition have increased, due to the wide availability of databases worldwide. We are currently facing significant challenges in pattern recognition, which is concerned with the automatic discovery of patterns in data through computer algorithms to take actions such as classifying the data into different categories [1]. Classification is fundamental when a data repository contains samples that can be used as the basis for later decision making [2]. Indeed, classification algorithms that aim at producing learning models from labeled training datasets are part of the supervised learning approach and are an essential component of several systems, such as speech recognition, handwritten symbol recognition, and data mining [3].

Many conventional classification algorithms were proposed in the long history of machine learning, some of which have been acknowledged as being highly accurate, particularly support vector machines (SVM) and random forests (RF). Furthermore, new classifiers are continuously being proposed, due to the complex nature and increasing scale of many real-world problems in different domains. For instance, deep learning (DL) [4] is a trending classification technique, and it is currently the state-of-the-art in machine learning research. It has demonstrated outstanding performance in image and speech recognition and related applications [5]. Despite these advantages, DL presents a significant challenge to acquiring a large amount of data, choosing the complex network structure, parameter settings, and interpretability results. Indeed, using DL with small sample datasets is a challenge [6,7]. Furthermore, DL makes the transfer of knowledge between experts and non-experts more difficult.

On the other hand, people not related to machine learning seek easy, interpretable, but effective learning algorithms to be used in decision-making problems. That is the reason why the straight line segment (SLS) classifiers are introduced [8]; they are binary classifiers based on distances between points and two sets of labeled straight-line segments. It is worthy of note that they are very light and can be embedded in small devices that use small memory.

SLS classifiers take advantage of some good characteristics of the two methods, learning vector quantization (LVQ) [9,10,11] and nearest feature line (NFL) [12,13], such as lower computational complexity, compared to support vector machines (SVM) [14]. Additionally, in [8,14,15], Ribeiro and Hashimoto showed that SLS classifiers are excellent alternatives to real applications, and their performance are comparable with SVM in binary classification problems. The preliminary results and academic collaborations outlined the SLS classifier as a good option, being competitive with well-known conventional classifiers for binary classification problems. The crucial part of SLS classifiers is their training phase. It consists of incrementally adjusting labeled straight-line segments to represent as much as possible “portions” of the feature space containing subsets of training points that all have the same label so that a new test point receives the same label of the straight-line segment nearest to it. In this way, it is expected that the final positions of the labeled straight-line segments provide the minimum (local) value for the mean squared error (MSE) of the training dataset. Thus, the problem of finding the final right place of straight-line segments is an optimization problem, which is solved by using a descent gradient. However, during the training phase, the straight-line segment lengths can grow significantly, giving a negative impact on the classification rate. In this work, we address this issue by solving the optimization problem in a constrained search space (tuned by a scale factor parameter) in order to restrict the straight-line segment lengths.

The rest of this paper is organized as follows: in Section 2, we briefly describe supervised learning methods based on distances and some related classifiers. Then, in Section 3, we present definitions and the learning algorithm of the straight-line segment classifiers. Later, Section 4 details the principal contribution of this work. Results for artificial and public datasets are presented in Section 5, while the discussion is listed in Section 6. The paper ends with conclusions and future perspectives in Section 7.

## 2. Supervised Learning Based on Distances

Supervised learning is an important component of all kinds of applications, such as speech recognition, handwritten symbol recognition, and data mining, among others [1,16]. It is one subfield of machine learning that learns from samples and generalizes to unseen cases. Thus, the aim of this kind of learning, and specifically in binary classification, is to build a concise predictive model of labels for two classes, generally using function $f:R^{d}\to \left\{0,1\right\}$ from a known dataset, represented by pairs of examples ($E_{n}=\{\left(x_{i},y_{i}\right)\in R^{d}\times \left\{0,1\right\}:i=1,2,\;$…,n}). The goal is to establish decision boundaries in the feature space that divide the patterns into its respective classes. Then, it is considered an error when the machine assigns a different label from the tutor [15].

Different methods and approaches have been proposed to overcome the two-class classification problem. Among the most frequently used, we can find the linear classifiers, neural networks, Bayesian networks, random forest, and support vector machines (SVM) [17]. However, the straight-line segment classifier is outlined as an exciting option, based on the preliminary results and academic collaborations [15,18,19,20]. The main contribution of the straight-line segment classifier is to introduce a classifier based on distances between a set of points and two sets of straight-line segments [15], where the extremities of the line segments do not need to be examples.

### 2.1. Related Classifiers

#### 2.1.1. K-Nearest Neighbor Classifier (k-NN)

**Figure 1:**
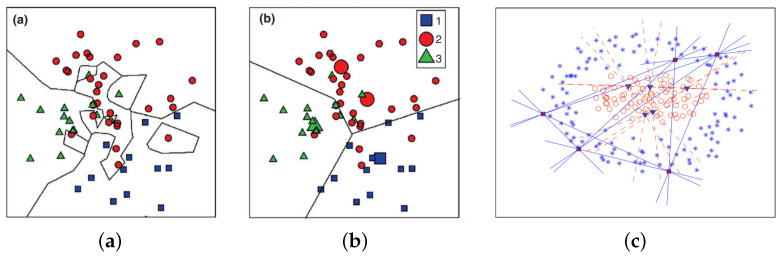
Example of a separable problem in 2D space. Decision boundaries obtained by (**a**) k-NN classifier; (**b**) LVQ, prototypes are marked by larger symbols; (**c**) NFL, feature line spaces drawn from 5 points. Figures extracted from (**a**,**b**) [23] and (**c**) [12].

Proposed by [21], the k-NN classifier is a simple algorithm that stores all available cases and classifies new cases based on a similarity measure. Ref. [22] demonstrates that the k-NN error is not more than twice the Bayes error, asymptotically. An example is classified by a majority vote of its neighbors and labeled to the most common class among its *k* nearest neighbors (see Figure 1a).

#### 2.1.2. Learning Vector Quantization (LVQ)

LVQ is one of the most powerful approaches for prototype-based classification of vector data [9]. The prototype adaptation scheme is based on attraction and repulsion during the learning [24]. In the case of supervised vector quantization, the prototypes are used to determine the classification decision. As can be seen in Figure 1b, the winning prototype is moved closer to the example feature vector if they share the same label, and it moves away otherwise [23].

#### 2.1.3. Nearest Feature Line (NFL)

The NFL is applicable where there are at least two prototypes for each class; it was proposed by [13]. This method uses a linear model to interpolate and extrapolate each pair of prototype feature points belonging to the same class. More specifically, the two prototype feature points are generalized by the feature line (FL), a straight line passing through the two points in the feature space (see Figure 1c). Hence, it virtually provides an infinite number of prototype feature points of the class, extending the capacity of the prototype. Finally, the classification is done by calculating the minimum Euclidean distance between the feature point and its projection to the feature line [12].

## 3. The Straight-Line Segment Classifier

In this section, we present the straight-line segment classifier (SLS classifier), whose main contribution is to introduce a binary classifier based on distances between a set of points and two sets of straight-line segments [15], where the extremities of the line segments are not necessarily part of the examples. In order to achieve this objective, in this section, we include its basic definitions and training algorithm.

### 3.1. Notation and Definitions

A straight-line segment with extremities *p* and *q* $\in R^{d+1}$ is defined as follows:

(1) $$L_{p,q}=\left\{x\in R^{d+1}:x=p+\lambda \cdot \left(q-p\right),0\leq \lambda \leq 1\right\}$$

**Figure 2:**
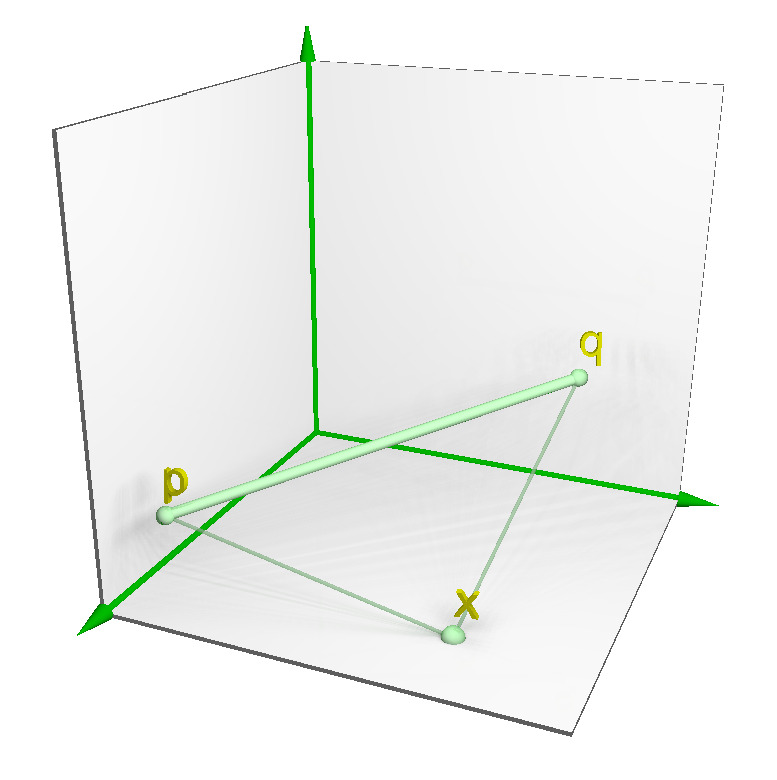
Representation of the distance between a point *x* and a straight-line segment with extremities *p* and *q* $\in R^{d+1}$.

Given a point $x\in R^{d}$, an extension of *x* to $R^{d+1}$ is denoted by $x_{e}=\left(x,0\right)$, adding one more coordinate with zero value. Moreover, a *pseudo-distance* between a point $x\in R^{d}$ and a straight-line segment $L_{p,q}\subseteq R^{d+1}$ is defined in Equation (2), where dist(a,b) denotes the Euclidean distance between two points $a,b\in R^{d+1}$; as can be seen in Figure 2. It is worth mentioning that this metric does not compute the Euclidean distance between a point (*x*) and a straight-line segment (*L*). However, it satisfies the following axioms [15]: (i) if p=q, then distP(x,L)=dist(x,p)=dist(x,q); (ii) if x∈L, then distP(x,L) is zero; and (iii) if x∉L, then distP(x,L) is greater than zero. Therefore, the farther *x* is from *L*, the greater distP(x,L).

(2) $$distP\left(x,L\right)=\frac{dist\left(x_{e},p\right)+dist\left(x_{e},q\right)-dist\left(p,q\right)}{2}$$

**Figure 3:**
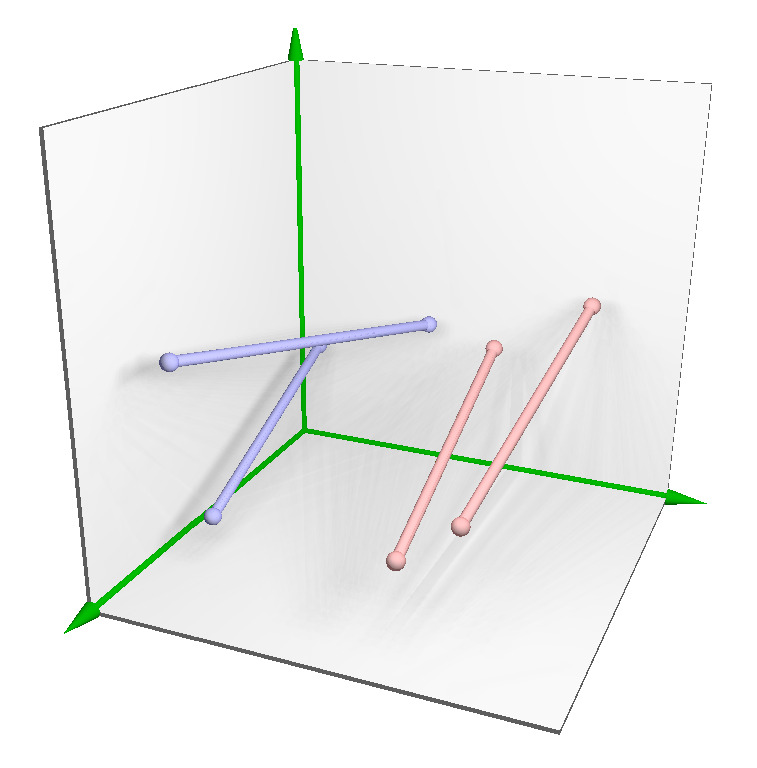
Representation of two sets of straight-line segments in color red and blue.

Since the SLS classifier is based on two sets of straight-line segments (red and blue, see Figure 3), a set of SLSs L is defined in Equation (3), where *m* represents the number of straight-line segments for each class.

(3) $$L=\{L_{p_{i},q_{i}}:p_{i},q_{i}\in R^{d+1},i=1,\ldots ,m\}$$

Furthermore, the discriminative function is defined in Equation (4), where $x\in R^{d}$ and ε is a small positive constant to avoid division by zero. Followed by the classification function denoted in Equation (5), where $S_{L_{0},L_{1}}\left(x\right)$ is a sigmoid function (see Equation (6)), where *g* is a real positive constant, which influences the slope of the sigmoid function. The larger the values of *g*, the more the sigmoid function approximates to a step function. It is worth mentioning that this value is optimized during the training phase.

(4) $$T_{L_{0},L_{1}}\left(x\right)=\sum_{L\in L_{1}}\frac{1}{distP(x,L)+\varepsilon }-\sum_{L\in L_{0}}\frac{1}{distP(x,L)+\varepsilon }$$

(5) $$\begin{array}{cc} F_{L_{0},L_{1}}\left(x\right) & \left\{\begin{array}{cc} 0, & if\;\;S_{L_{0},L_{1}}\left(x\right)\;<\;0.5;\\ 1, & \mathrm{otherwise} \end{array}\right. \end{array}$$

(6) $$S_{L_{0},L_{1}}\left(x\right)=\frac{1}{1+e^{-g(T_{L_{0},L_{1}}\left(x\right))}}$$

### 3.2. Training Algorithm

As stated in [25] “supervised statistical learning involves building a statistical model for predicting or estimating an output based on one or more inputs by reducing the error on a training data set”. Therefore, as described in [15], given a set of *n* examples $E_{n}=\left\{\left(x_{i},y_{i}\right)\in R^{d}\times \left\{0,1\right\}:i=1,2,\ldots ,n\right\}$, the objective of the supervised learning algorithm of the SLS classifier is to find two sets of straight-line segments (SLSs) ($L_{0}$ and $L_{1}$), based on the fact that points *x* closer to $L_{0}$ (or $L_{1}$) and farther from $L_{1}$ (or $L_{0}$) (i) lead the classification function $F_{L_{0},L_{1}}\left(x\right)$ to 0 (or 1) and (ii) minimize the mean squared error (Equation (7)), which is a differentiable function.

(7) $$MSE\left(F_{L_{0},L_{1}}\right)=\frac{1}{n}\sum_{i=1}^{n}{\left[S_{L_{0},L_{1}}\left(x_{i}\right)-y_{i}\right]}^{2}$$

**Figure 4:**
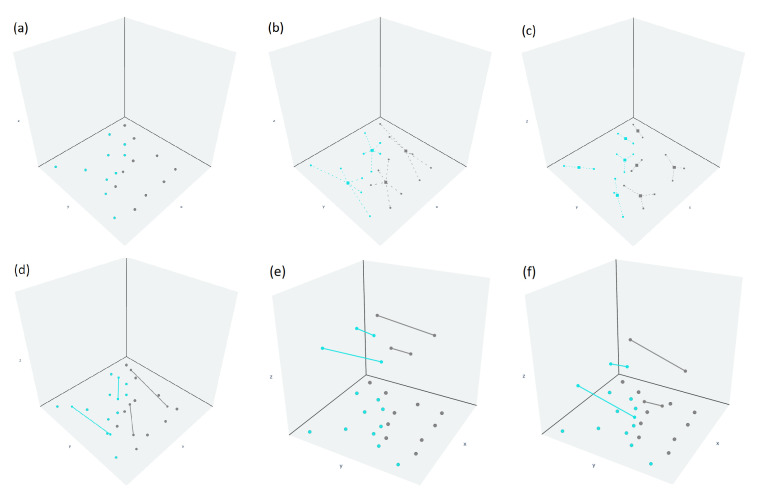
Training algorithm steps: placing (**a**–**d**) and tuning (**e**–**f**). Modified from [15].

As proposed in [14], the training algorithm is composed of two phases, as depicted in Figure 4 and detailed in Algorithm 1.
**Algorithm 1:** Training algorithm**Require:**$E_{n}=\{\left(x_{i},y_{i}\right)\in R^{d}\times \left\{0,1\right\}:i=1,2,\ldots ,n\}\;m\;=\;numSLS,\;l\;=\;1,\;g\;=\;1,$       gdParams = $\left[I_{min},I_{max},\gamma_{inc},\gamma_{dec},Disp_{min},R_{min}\right]$**Ensure:**$g,\;L_{0},\;L_{1}$     {Placing Phase}1: $X_{0}\leftarrow \{\left(x_{i},y_{i}\right)\in E_{n}$ and $y_{i}=0\}$2: $X_{1}\leftarrow \{\left(x_{i},y_{i}\right)\in E_{n}$ and $y_{i}=1\}$3: **for**
*class* ← **to** 1 **do**4: $\;\;\left(c_{class}^{0},\ldots ,c_{class}^{m-1}\right),\left(C_{class}^{0},\ldots ,C_{class}^{m-1}\right)\leftarrow$ KMeans($X_{class}$,*m*)5:     **for**
*z* ← 0 **to**
*m* − 1 **do**6: $\;\;\;\;\;\;\left(d_{class}^{0},d_{class}^{1}\right),\left(D_{class}^{0},D_{class}^{1}\right)\leftarrow$ KMeans($C_{class}^{z}$,2)7: $\;\;\;\;\;\;\;p_{e}\leftarrow \left(d_{class}^{0},l\right)$8: $\;\;\;\;\;\;\;q_{e}\leftarrow \left(d_{class}^{1},l\right)$9:         add $\left(p_{e},q_{e}\right)$ to $L_{class}$10:     **end for**11: **end for**     {Tuning Phase}12: g←113: $\alpha \leftarrow [g,L_{0}$, $L_{1}]$14: $[g,L_{0}$, $L_{1}]\leftarrow$ GradDesc(α,gdParams)

#### 3.2.1. Placing

This phase consists of pre-allocating (finding the initial positions of) the straight-line segments (in $L_{0}$ and $L_{1}$), as described in Algorithm 1 from line 1 to 11, based on the fact that points *x* closer to $L_{0}$ (or $L_{1}$, respectively) and farther from $L_{1}$ (or $L_{0}$, respectively) lead the classification function $F_{L_{0},L_{1}}\left(x\right)$ to 0 (or 1, respectively). To achieve this goal, the set of examples $E_{n}$ is divided into two groups: $X_{i}=\left\{x\in R^{d}:\left(x,y\right)\in E_{n}\;\mathrm{and}\;y=i\right\}$ (for i=0,1). Then, the clustering algorithm *k*-means is applied to each group, with k=m, where *m* represents the number of SLSs required per class. As can be seen in Algorithm 1 line 4, k-means returns two sets: (i) the centroids *c* and the points belonging to an specific cluster *C*. Later, with the objective to obtain the initial extremities of the SLSs ($p_{i}$ and $q_{i}$) for each cluster, the *k*-means algorithm (with k=2) is applied again, but at this time, to each cluster obtained from the previous *k*-means application.

#### 3.2.2. Tuning

The purpose of this phase is to minimize the mean square error function, as described in Algorithm 1 from lines 12 to 14. Therefore, to accomplish this task, the gradient descent technique [1] is used to find the final positions of the SLSs in $L_{0}$ and $L_{1}$ (which contains the initial positions obtained in the previous phase) and the value of *g* (sigmoid inclination; see Equation (6)), whose default value is 1 such that the derivative of the mean square function is equal to zero.

As detailed in [20], this classifier version differs from the original in a sign change of the MSE derivative concerning the parameter *g*, which defines the sigmoid “smoothness” (Equation (6)). Then, the application of the gradient descent technique, which occurs only once, including the value of *g* in the parameters vector, adjusts its value during optimization in conjunction with the straight-line segment final positions (see Algorithm 1 line 14). Lastly, the stop criterion depends on the gradient’s Euclidean norm, specifically when it has reached a specific small predefined value ($Ng_{tolerance}$). If $\left\|\nabla MSE(S_{L_{0},L_{1}})\right\|$ is small enough, it can be approximated to zero, meaning that it is close to the optimal solution. Either, if $MSE(S_{L_{0},L_{1}})$ is convex, the gradient is monotonous and continuous, so if it is close to zero, it is close to the minimum. Despite the fact that the gradient descent method does not guarantee the global minimum and the final solution (positions of the SLSs) depends on the initial placing phase, it is successfully applied.

## 4. Bounding Box Approach for Straight-Line Segments Growth Restriction

**Figure 5:**
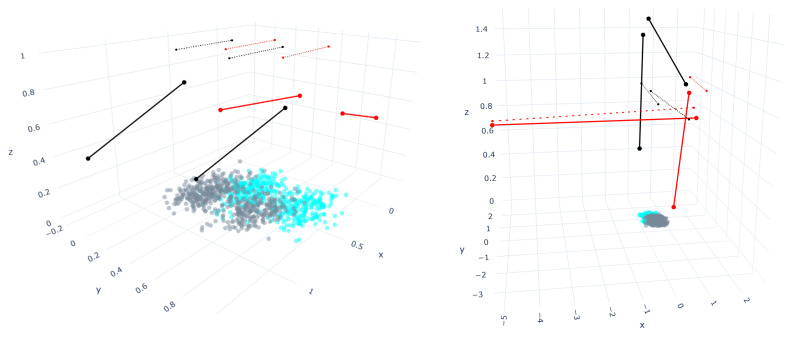
Representation of the initial (dashed line) and final (solid line) positions of the straight-line segments for the S-Shape distribution using 2-2 straight-line segments per class. In the plots, the red color lines belong to $class_{1}$ and the ones in black, to $class_{0}$.

The length of the straight-line segments has a meaningful influence on the classification rate of the SLS classifier. The straight-line segments are displaced in the space during the training to achieve a minimum mean squared error by applying the gradient descent method. Nevertheless, most of the time, it is not just a displacement but also a growth of the straight-line segments to represent more points and obtain a better classification rate. According to Medina et al. [20], in some cases, the straight-line segments can grow significantly. Although it leads to high classification rates, visually, the segments are apart from the training set. As shown in Figure 5, the left side depicts the results (79.45%) after using the k-means algorithm to find the initial positions. However, the right side represents a set of straight-line segments that visually are far from the dataset and also achieve a correct classification (79.32%) similar to the left side. It is worth mentioning that the initial positions were randomly initialized.

**Figure 6:**
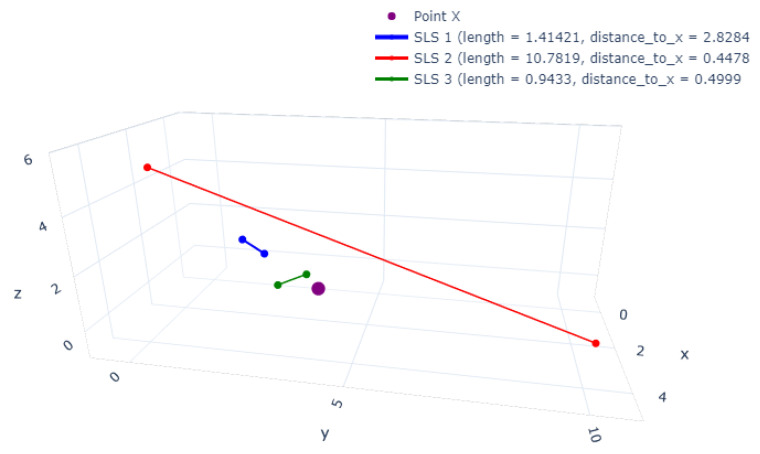
Distance comparison between one fixed point X (in purple) and three straight-line segments (i) in blue, a short length and far from point X; (ii) in green, a short length and close to the point X; and, (iii) in red, long length and far from point X.

In order to understand this behavior, we explore the pseudo-distance, which represents the distance from a point to one straight-line segment. We hypothesized that by definition, Equation (2) has a disadvantage: the distance between a point to a distant and long straight-line segment could be less than the distance between the same point to a short and close straight-line segment. Therefore, to prove our hypothesis, in Figure 6, we plot the distance from the red straight-line segment, which has a length of 10.78 and the green one with the length of 0.94. For these cases, the distances are 0.4478 and 0.4999, respectively. These results prove our hypothesis that the distance from SLS-2 (red and long) is less than from the SLS-3 (green and short).

Given this context, we analyze this behavior at the training phase. Then, we can assume that for each iteration of the gradient descent, the length of a long straight-line segment could keep growing because the distance computed according to Equation (2) is short. Moreover, it could lead to problems of misclassification and the overlapping of straight-line segments from different classes. Therefore, to represent the training points with two sets of straight-line segments without exceeding a certain length, we propose an approach to restrict the straight-line segments’ growing space, based on the idea of the *bounding box*, which is defined as the rectangle that is just large enough to contain all objects. In this paper, we define a rectangular box that encloses the straight-line segments, restricting their length. This bounding box is determined by each minimum and maximum coordinate value as defined in Equation (8).

(8) $$\left[\left\{min_{i},\ldots ,min_{d},min_{d+1}\right\}\left\{max_{i},\ldots ,max_{d},max_{d+1}\right\}\right]\in R^{d+1}:i=1,2,\ldots ,d,d+1;$$

**Figure 7:**
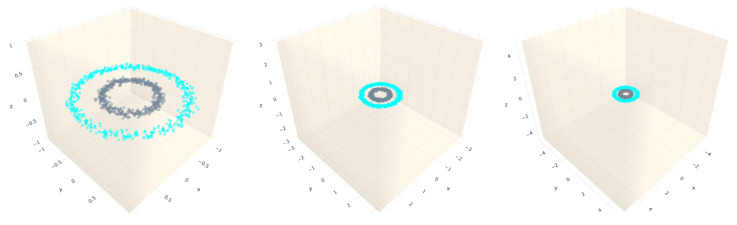
Proposed bounding boxes for restricting the straight-line segments’ growing space, using scale factors (mbb_factor): 0.0, 2.0 and 4.0, respectively.

It is worth mentioning that we introduce a new coordinate d+1 with value 1 at the tuning phase (see Section 3.2.2). Thus, we fix the minimum and maximum value of that coordinate to {−1,1}. An example of an increment in the bounding box size for the circles distribution is depicted in Figure 7. In order to scale the bounding box, we use a variable called mbb_factor, which increments the minimum and maximum values by a percentage of each coordinate in the following way:

$$\begin{array}{ccc} NewMin_{i} & = & min_{i}+\left(min_{i}*mbb\_factor\right)\\ NewMin_{d+1} & = & -1+(-1*mbb\_factor)\\ NewMax_{i} & = & max_{i}+\left(max_{i}*mbb\_factor\right)\\ NewMax_{d+1} & = & +1+(1*mbb\_factor) \end{array}$$

Finally, the minimum bounding box is used as a stop criterion for the gradient descent besides the Euclidean norm and a maximum number of iterations. Therefore, the gradient descent will also stop whenever any of the extremities from the straight-line segments exceed the bounding box. Then, line 14 of Algorithm 1 is replaced with $[g,L_{0}$, $L_{1}]\leftarrow$ GradDesc(α,gdParams,minimumBoundingBox).

## 5. Results

This section describes the artificial datasets built for this work and the eight public datasets used for testing the proposed approach. Their configuration, including the classifier parameters, number of examples, and training times, are also described. Finally, the obtained results and a comparison between a previous version of the classifier and the proposed one is shown.

### 5.1. Artificial Datasets

Artificial datasets are useful for a better understanding of the behavior of algorithms in response to different hyperparameters. They should meet some properties, such as the following: quick and easy generation and visualization in two dimensions; having known outcomes for comparison with predictions; being stochastic, allowing random variations on the same problem; and scalability.

**Figure 8:**
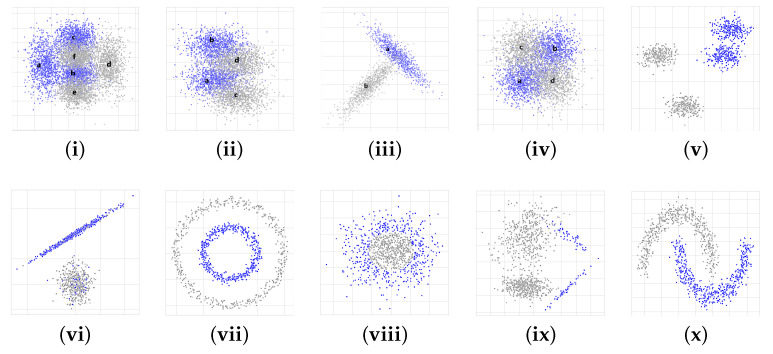
Artificial dataset types used in our research. On the first row: (**i**) F-Shape, (**ii**) S-Shape, (**iii**) Simple-Shape, (**iv**) X-Shape, and (**v**) Blobs. On the second row: (**vi**) Blobs with Noise, (**vii**) Circles, (**viii**) Gaussian, (**ix**) Imbalanced, and (**x**) Moon. On each graph, $class_{0}$ is represented by gray color and $class_{1}$ by blue color.

Given this context, we used four distributions, renamed for the interest of this research to (i) F-Shape, (ii) S-Shape, (iii) Simple-Shape, and (iv) X-Shape, proposed by [14]. As depicted in Figure 8i–iv, for each class C∈{0,1} in $R^{2}$, the distributions were designed according to probability distributions divided into several regions. Each region is associated to a density function defined by the sum of *M* normal two-dimensional functions denoted by Equation (9), where $\mu_{i}^{C}\in R^{2}$ is the center of the normal density function, $\Sigma_{i}^{C}$ is the 2×2 covariance matrix, and $P^{C}$ is a real number such that $\sum_{i=1}^{M}P_{i}^{C}=1$ whose values are detailed in Table 1. Additionally, Ribeiro and Hashimoto [15] applied the Bayes classifier [16] to obtain the ideal classification rate for each distribution (see Table 2) since the probability density function is known.

(9) $$p\left(x,y=C\right)=\sum_{i=1}^{M}P_{i}^{C}Normal\left(x,\mu_{i}^{C},\Sigma_{i}^{C}\right)$$

Furthermore, six new dataset types (see Figure 8v–x) were considered, and to generate samples for them, we used a data generator from the scikit-learn package [26] with the parameters detailed in Table 3, briefly described below:

(v) Blobs: it consists of two blobs from a Gaussian distribution for each class (gray and blue colors, respectively).
(vi) Blobs with noise: they are composed of one blob for each class. The label of 20% of the samples from each blob is randomly exchanged.
(vii) Circles: they are a sample that falls into two concentric circles.
(viii) Gaussian quantiles: they are constructed by taking a multi-dimensional standard normal distribution and defining classes separated by nested concentric multi-dimensional spheres. Each class has an equal number of instances.
(ix) Imbalanced: it contains the number of gray instances $class_{0}$ that significantly outnumbers (nine times) the blue instances $class_{1}$, which leads to class imbalance.
(x) Moon: it is composed of two half moons, one for each class.

For comparison purposes, we estimated the naive Bayes classifier [27] accuracies for each dataset type. To that end, for each dataset type (including the four original ones), we generated eleven datasets, each one containing 160,000 sampling points. Then, we trained ten naive Bayes classifiers [27] for the first ten datasets, and computed their accuracies using the last one. The results can be seen in Table 4. It is worth mentioning that we achieved a low classification rate for the X-Shape distribution (logical XOR) with 49.31% because of the class conditional independence assumption of the naive Bayes classifier model; that is, given a class, a feature variable is independent of any other feature variable. Moreover, there is a difference of 4% concerning the optimal Bayes classifier accuracy rate on the four distributions proposed in the original work.

### 5.2. Experiments

**Figure 9:**
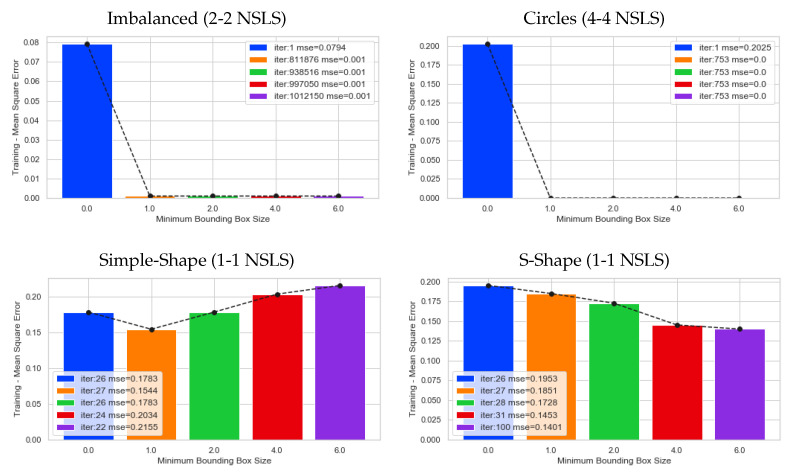
Number of iterations at gradient descent algorithm stops when using different bounding box scale factors.

Based on the approach proposed in Section 4, some experiments over ten artificial datasets, using the parameters detailed in Table 5, were performed to find out if delimiting the growth of the straight-line segments improves the classification rate of the SLS classifiers. Figure 9 shows the mean squared error (Equation (7)) achieved when the training algorithm stops after one of the straight-line segments falls out of the bounding box.

**Figure 10:**
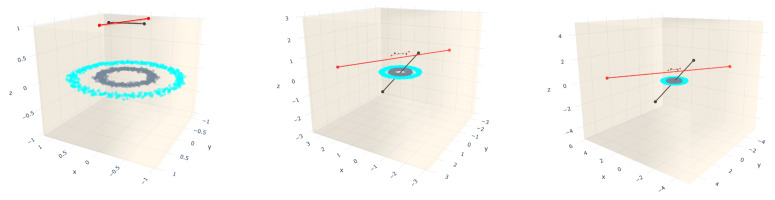
A 3D visualization of the final straight-line segment positions obtained after applying the training algorithm, using one straight-line segment per class. Each column represents a different value of mbb_factor = {0,2,4} with accuracies of 73.35%, 99.9% and 99.9%, respectively.

For instance, for the imbalanced dataset, using 2-2 SLS per class, we can see that one of the extremities of the straight-line segments fell out of the bounding box with scale factor is equal to 6.0 at iteration 1,012,150, in contrast to the situation when the bounding box is not scaled, where the algorithm stops at the first iteration. The circles dataset presents the same behavior regarding the bounding box scale. Moreover, for visualization purposes, in Figure 10, we display the initial (dashed lines) and final positions (solid lines) of the straight-line segments before they fall out of the bounding box applied to the circles dataset. Additionally, for both datasets, the MSE is very close to zero. Therefore, from these two plots, we can conclude that a bigger bounding box will take many iterations to stop the training algorithm because it needs an extremity of any straight-line segments to be out of the bounding box. However, due to the randomness feature of the k-means used to find the initial positions, this affirmation is not always true, as shown in Figure 9 (second row). For the datasets presented in this row, the training algorithm stops earlier than expected. However, for the datasets in the first row of the same figure, the number of iterations increases, while the scale factor increases. Unlike the datasets presented in the second row, the iterations are less than 100 when the scale factor is equal to 6.0, which means that the straight-line segments grow faster and fall out of the box quickly.

From the results, we can notice two behaviors of the training algorithm: (i) it stops when one of the extremities of any straight-line segment falls out of the bounding box; or (ii) it has to be interrupted manually because of countless iterations caused by the fact that the straight-line segments never fall out of the large size of the bounding box.

**Figure 11:**
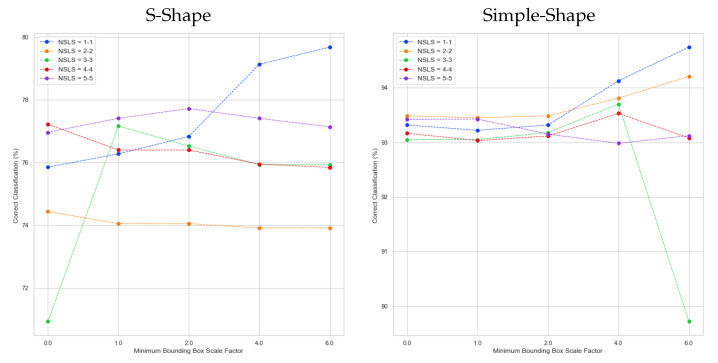
Correct classification at test phase for distributions S-Shape and Simple-Shape using from 1 to 5 straight line segments per class and 0, 1, 2, 4, 6 as bounding box scale factors.

Additionally, several experiments were performed with different bounding box scale factors, such as 0, 1, 2, 4 and 6, using from 1 to 5 straight line segments per class (see Figure 11). From these experiments, we extracted two charts, which show the classification rates achieved by two distributions: S-Shape and Simple-Shape (see Figure 11). As can be seen, on one hand, the bounding box generated (scale factor = 0.0) is too small, and it does not let the straight-line segments move in the space. On the other hand, when mbb_factor = 4.0 and 6.0, the bounding boxes are too big; consequently, the algorithm takes too many iterations since the straight-line segments never fall out of the bounding box. Nonetheless, the classification rates have high values, which could be due to the disadvantage of the pseudo distance function detailed in Section 4. In addition, these plots show that the classification rates increase for scale factors from 0.0 to 2.0 and remain the same or similar for higher values of scale factors.

### 5.3. Public Datasets

In order to demonstrate the feasibility, robustness, and performance of the proposed classifier on real problems, eight public datasets described in Table 6 were extracted from the UCI Machine Learning Repository [28]. The experiments were conducted according to the following specifications:

- Pre-processing: each dataset is normalized between −1 and 1, and the mean of the column replaces the missing values.
- Validation: stratified dataset split, preserving the percentage of samples for each class (train 75%, test 25%); and 10-fold cross validation where the training dataset is divided into 10 stratified folds.
- Number of straight-line segments: from 1 to 10 for each class, obtaining 100 models.
- Model selection: exhaustive search to find the straight-line segments’ best configuration.
- Gradient descent parameters: $\gamma_{init}$ = 0.1; $\gamma_{inc}$ = 0.1; $\gamma_{dec}$ = 0.5 and $Ng_{tolerance}$ = 0.001.
- The classifier was implemented in C++ using Armadillo library for Linear Algebra & Scientific Computing [29,30].
- All the experiments were performed in a 64-bit computer with 12 cores of 3.60 GHz under the Ubuntu/Linux operating system.

Since we applied an exhaustive model selection approach on the experiments, in Table 7, we show the classification accuracy rates obtained for each scale factor (1.0, 1.5 and 2.0) based on the mean squared error computed for the validation and test sets. In the same table, we detail the number of straight-line segments per class used for achieving those classification rates. As can be seen, in five datasets, the best result was achieved using 2.0 as a scale factor for the bounding box.

Moreover, shown in Table 8, we compared the original classifier [15] and the one proposed in this paper. We also performed an exhaustive search for the number of straight-line segments per class used by the original classifier for this experiment. The results presented in Table 8 show that the accuracies achieved by the proposed classifier using an exhaustive model selection (number of straight-line segments per class and the bounding box scale factor) outperform the original one by several percentage points. Regarding the number of straight-line segments, the proposed classifier uses fewer than the original one. It is worth mentioning that the original classifier always uses the same number of straight-line segments per class.

Furthermore, Table 8 also shows a comparison between the results of the proposed SLS classifier and the ones from other learning algorithms such as neural networks and the support vector machines. These results were extracted from [39], which evaluates 179 classifiers arising from 17 families, and applied to 121 datasets (representing the whole UCI data, excluding the large-scale problems). The classifier most likely to be the best, according to Fernandez-Delgado et al. [39], is the random forest. However, the difference is not statistically significant with the second best—the SVM with Gaussian kernel implemented in C using LibSVM (Ionosphere and Sonar dataset accuracy were extracted from: http://fizyka.umk.pl/kis-old/projects/datasets.html (accessed on 13 October 2021)). Table 8 also presents the results obtained using the SLS classifier with three variants: (i) the original algorithm, (ii) a previous version of the classifier without the bounding box approach, and (iii) our proposal with the best bounding box scale factor founded in the experiments. It is worth mentioning that Table 8 also presents a comparison with neural networks (NNs) which are a kind of simplified version of deep learning (DL) network. In fact, DL networks can be built by using NNs with higher complexity. Since we are dealing with UCI public datasets with a number of samples much smaller than the number of samples expected to be used in DL classifiers, the fairest comparison should use a kind of simpler version of DL networks.

## 6. Discussion

**Figure 12:**
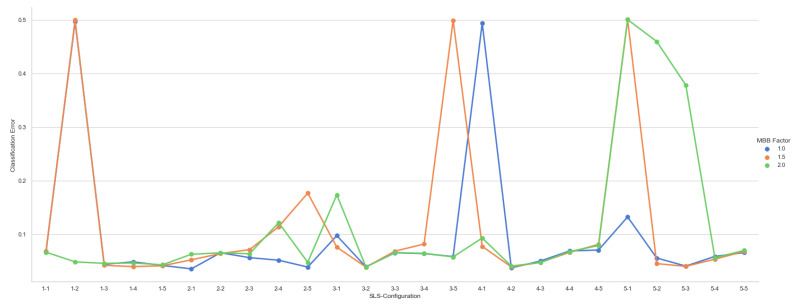
Classification errors at testing phase for the Simple-Shape distribution using {1.0,1.5,2.0} as scale factors for the bounding box.

The straight-line segment length was an existing problem for the straight-line segment classifier, specifically when the k-means algorithm does not find optimal initial positions, and the gradient descent algorithm allows their length to grow faster, making the straight-line segments distant from the training dataset samples. Therefore, this paper proposes an approach to constrain its growing space by defining a minimum bounding box for the straight-line segments. The user defines a scale factor that allows the growth of the bounding box. Several experiments changing the scale factor value were performed on artificial datasets, concluding that using a scale factor bigger than 2.0 leads to lower classification rates and a higher number of iterations, and consequently, increasing the computational cost. For instance, in Figure 12, we can see the classification error curves for SLS classifiers, using 1–5 straight-line segments per class and three suggested bounding box scale factors. As can be seen, the curves show the same behavior when increasing the number of straight-line segments. Therefore, the greater the number of straight-line segments, the lower the classification error and, consequently, the higher the classification rate. In addition, it can also be seen that using 2.0 as a scale factor for the bounding box leads to a higher classification errors than the ones obtained with a bounding box scale factor equal to one.

Several works in the literature describe which classifiers are more suitable to tackle a diversity of applications. Despite the long tradition of pattern recognition research, no technique yields the best classification in all scenarios [40]. From the results in Table 8, we can conclude that our proposed method achieves acceptable results when compared with other classifiers. Since the results in the column “All” include a list of 179 conventional machine learning classifiers from which the random forest frequently achieves the best results, we can see that our proposal outperforms these results in half the cases. It is worth mentioning that in none of the cases, the classifier achieves the worst classification rate. Moreover, when comparing our proposal considering the standard deviation, we achieve similar classification rates than the best classifiers analyzed in [39]. In addition, Table 8 shows a column “NN”, which contains the best results obtained by using NNs (a simplified version of DL networks), reported by Fernandez-Delgado et al. [39]. NNs achieved the highest classification rate only for the Sonar dataset. Among the results from the SLS classifiers, we can conclude that using the bounding box approach improves the classification rates, achieving the highest rate in most cases. It even improves the results from the previous version of the SLS classifier, which only stops when the maximum number of iterations is reached (1000 iterations).

## 7. Conclusions

This paper aims to provide a method for adjusting the straight-line segments in a constrained search space to build reliable SLS classifiers. To that end, we illustrated our approach based on a bounding box by using simulated and public datasets. From the results, we can conclude that our way of increasing the bounding box could be improved by considering the data variance into the scale factor calculations. An alternative to the straight-line segments growth restriction must be explored to constrain the length of each straight-line segment during the training algorithm with the gradient descent method. Moreover, a different model selection approach should avoid the exhaustive search of parameters, including the minimum bounding box scale factor in the model selection. This extension could help find the best model among all the possible parameters. Furthermore, people unrelated to the machine learning field are searching for easily understandable and interpretable but accurate classifiers to be used in decision-making problems. The straight-line segment classifier emerges as an interesting alternative with low complexity and computational costs, and easy results interpretation, compared to the complex deep learning network structure design, parameter settings, and interpretability, making it attractive to be embedded in small devices. The SLS classifier would make more effortless the transfer of knowledge between experts and non-experts.

## Figures and Tables

**Table 1:** Set of values for the probability density functions used for the generation of the artificial distribution. Adapted from [20].

Dist.	Class (C)	Mean (μ)	Covariance (Σ)	Prob(*P*)	Label
F-Shape *M* = 3	0	[0.125,0.5]	[0.01,0,0,0.04]	0.574	(a)
		[0.5,0.375]	[0.012,0,0,0.01]	0.213	(b)
		[0.5,0.875]	[0.012,0,0,0.01]	0.213	(c)
	1	[0.875,0.5]	[0.01,0,0,0.04]	0.574	(d)
		[0.5,0.125]	[0.012,0,0,0.01]	0.213	(e)
		[0.5,0.625]	[0.012,0,0,0.01]	0.213	(f)
S-Shape *M* = 2	0	[0.4,0.4]	[0.02,0,0,0.01]	0.5	(a)
		[0.4,0.8]	[0.02,0,0,0.01]	0.5	(b)
	1	[0.6,0.2]	[0.02,0,0,0.01]	0.5	(c)
		[0.6,0.2]	[0.02,0,0,0.01]	0.5	(d)
Simple-Shape *M* = 1	0	[0.6,0.6]	[0.01,−0.009,−0.009,0.01]	1	(a)
	1	[0.4,0.4]	[0.01,0.009,0.009,0.01]	1	(b)
X-Shape *M* = 2	0	[0.25,0.25]	[0.04,0,0,0.04]	0.5	(a)
		[0.75,0.75]	[0.04,0,0,0.04]	0.5	(b)
	1	[0.25,0.75]	[0.04,0,0,0.04]	0.5	(c)
		[0.75,0.25]	[0.04,0,0,0.04]	0.5	(d)

**Table 2:** Bayes classification accuracies reported in [15].

Distribution	Bayes Classifier (%)
F-Shape	91.43
S-Shape	84.39
Simple-Shape	96.95
X-Shape	81.11

**Table 3:** Set of *scikit-learn* parameters used to generate the six new artificial datasets.

Dataset Type	Parameters
Blobs	n_samples = 160,000, centers = 4, n_features = 2, random_state = 222
Blobs with Noise	n_samples = 160,000, n_features = 2, n_informative = 2, n_redundant = 0, n_repeated = 0, n_classes = 2, n_clusters_per_class = 1, class_sep = 2, flip_y = 0.2, weights = [0.5, 0.5], random_state = 222
Circles	n_samples = 160,000, factor = 0.5, noise = 0.05, random_state = 222
Gaussian Quantiles	n_samples = 160,000, n_features = 2, n_classes = 2, random_state = 222
Imbalanced	n_samples = 160,000, n_features = 2, n_informative = 2, n_redundant = 0, n_repeated = 0, n_classes = 2, n_clusters_per_class = 2, class_sep = 1.5, flip_y = 0, weights = [0.9, 0.1], random_state = 222
Moon	n_samples = 160,000, noise = 0.1, random_state = 222

**Table 4:** Mean and standard deviation of the correct classification rate obtained with the naive Bayes classifier.

Distribution	1st	2nd	3rd	4th	5th	6th	7th	8th	9th	10th	Mean	Stdev
Blobs	99.76	89.20	99.63	60.57	99.38	82.69	96.25	97.85	93.66	90.68	90.97	12.03
Blobs_noise	89.05	89.42	89.90	89.22	89.89	90.03	90.02	89.73	89.91	89.02	89.62	0.40
Circles	99.99	99.99	99.99	99.99	99.99	99.99	99.99	99.99	99.99	99.99	99.99	0.002
F-Shape	82.16	82.00	82.08	82.05	82.08	82.19	82.10	82.12	82.02	82.02	82.08	0.06
Gaussian	97.27	97.23	97.33	97.42	97.33	97.24	97.21	97.32	97.35	97.41	97.31	0.07
Imbalanced	98.37	99.66	99.74	98.89	98.77	98.26	98.75	97.97	95.56	99.25	98.52	1.19
Moon	87.92	88.03	87.95	88.02	87.93	88.02	87.99	88.02	88.03	87.98	87.99	0.04
S-Shape	79.27	79.57	79.26	79.52	79.53	79.59	79.60	79.60	79.44	79.52	79.49	0.13
Simple-Shape	92.37	92.36	92.47	92.39	92.35	92.41	92.33	92.33	92.35	92.40	92.38	0.05
X-Shape	50.35	47.44	47.36	47.43	49.73	49.91	47.52	50.36	55.72	47.29	49.31	2.62

**Table 5:** Set of parameters for the experiments performed with the artificial datasets.

Parameter	Values
Training Samples	10 samples of 1000 examples
Test Sample	160,000 examples
Validation	10-Fold-Cross-Validation
mbb_factor	0.0, 1.0, 2.0, 4.0, 6.0
Num. SLS per class	1-1, 2-2, 3-3, 4-4, 5-5
Gradient Descent-$\gamma_{init}$	0.1
Gradient Descent-$\gamma_{inc}$	0.1
Gradient Descent-$\gamma_{dec}$	0.5
Gradient Descent-$Ng_{tolerance}$	0.001

**Table 6:** Public datasets features.

Dataset	# Examples	Dim	Train-Size	Test-Size
Australian Credit Approval [31]	690	14	517	173
Breast Cancer Wisconsin [32]	683	10	512	171
Pima Indians Diabetes [33]	768	8	576	192
German Credit Data [34]	1000	24	750	250
Heart [35]	270	13	202	68
Ionosphere [36]	351	34	263	88
Liver Disorders [37]	345	6	258	87
Sonar Mines vs Rocks [38]	208	60	156	52

**Table 7:** Summary of the best models (number of straight-line segments per class and the bounding box scale factor) obtained after an exhaustive model selection approach from 1 to 10 straight-line segments per class for each bounding box scale factor, evaluating the mean squared error on validation and test datasets.

Dataset	The SLS Best Configuration	MSE-Validation (%) (1.0, 1.5 and 2.0)	MSE-Test (%) (1.0, 1.5 and 2.0)
Australian	{1-1, 2-3, **1-1**}	{89.22%,88.85%,88.77%}	{89.60%,89.80%,89.81%}
Breast Cancer	{8-10, 8-10, **8-10**}	{98.41%,98.41%,98.41%}	{94.72%,95.35%,95.65%}
Diabetes	{**3-2**, 7-2, 7-2}	{84.08%,83.77%,83.83%}	{83.22%,82.35%,82.96%}
German	{2-2, 1-1, **1-2**}	{82.50%,82.47%,82.45%}	{84.80%,84.36%,85.33%}
Heart	{**2-2**, 1-2, 1-1}	{87.27%,87.31%,87.16%}	{87.39%,85.17%,86.69%}
Ionosphere	{10-8, 10-8, **10-8**}	{96.98%,96.98%,96.98%}	{94.12%,94.35%,94.50%}
Liver Disorders	{5-2, 5-2, **5-2**}	{81.41%,81.41%,81.41%}	{75.51%,75.65%,76.59%}
Sonar	{5-7, **6-7**, 6-7}	{91.12%,91.10%,91.10%}	{89.38%,92.38%,90.04%}

**Table 8:** The best results between the proposed SLS classifier and other literature classifiers. The best results among them are highlighted in bold, and the standard deviation are presented in parentheses.

Dataset	All	NN	SVM	SLS Classifier						
	[39]	[39]	[39]	Original		Proposed w/o MBB		Proposed w/ MBB		
Australian	69.1	68.8	68.2	87.28	8-8	86.00 (1.49)	1-1	**89.81** (4.2)	1-1	*2.0*
Breast-Cancer	97.4	97.4	97.0	94.74	8-8	**98.20** (1.46)	8-10	95.65 (1.6)	8-10	*2.0*
Diabetes	79.0	78.1	78.3	73.44	7-7	76.79 (2.38)	3-2	**83.22** (3.5)	3-2	*1.0*
German	79.0	78.1	77.6	72.40	1-1	72.16 (0.48)	1-2	**85.33** (1.6)	1-2	*2.0*
Heart	**88.2**	86.9	88.1	80.88	6-6	78.42 (1.38)	2-2	87.39 (6.2)	2-2	*1.0*
Ionosphere	95.5	95.2	95.5	95.45	7-7	95.56 (3.53)	10-8	94.50 (2.0)	10-8	*2.0*
Liver-Disorders	-	-	-	72.41	5-5	72.08 (2.08)	5-2	**76.59** (7.5)	5-2	*2.0*
Sonar	**97.1**	**97.1**	90.4	88.46	7-7	88.57 (1.51)	6-7	92.38 (6.0)	6-7	*1.5*

## Data Availability

The data presented in this study are available on request from the corresponding author.
